# Designing electrode configuration of electroosmosis based edema treatment as a complement to hyperosmotic therapy

**DOI:** 10.1007/s00701-021-04938-5

**Published:** 2021-07-22

**Authors:** Teng Wang, Svein Kleiven, Xiaogai Li

**Affiliations:** Division of Neuronic Engineering, Department of Biomedical Engineering and Health Systems, KTH Royal Institute of Technology, Hälsovägen 11C, SE-141 52, Huddinge, Sweden

**Keywords:** Cerebral edema, Electroosmosis based edema treatment, Hyperosmotic therapy, Patient-specific head model, Electrode configuration

## Abstract

**Background:**

Hyperosmotic therapy is a mainstay treatment for cerebral edema. Although often effective, its disadvantages include mainly acting on the normal brain region with limited effectiveness in eliminating excess fluid in the edema region. This study investigates how to configure our previously proposed novel electroosmosis based edema treatment as a complement to hyperosmotic therapy.

**Methods:**

Three electrode configurations are designed to drive the excess fluid out of the edema region, including 2-electrode, 3-electrode, and 5-electrode designs. The focality and directionality of the induced electroosmotic flow (EOF) are then investigated using the same patient-specific head model with localized edema.

**Results:**

The 5-electrode design shows improved EOF focality with reduced effect on the normal brain region than the other two designs. Importantly, this design also achieves better directionality driving excess edema tissue fluid to a larger region of surrounding normal brain where hyperosmotic therapy functions better. Thus, the 5-electrode design is suggested to treat edema more efficiently via a synergic effect: the excess fluid is first driven out from the edema to surrounding normal brain via EOF, where it can then be treated with hyperosmotic therapy. Meanwhile, the 5-electrode design drives 2.22 mL excess fluid from the edema region in an hour comparable to the other designs, indicating a similar efficiency of EOF.

**Conclusions:**

The results show that the promise of our previously proposed novel electroosmosis based edema treatment can be designed to achieve better focality and directionality towards a complement to hyperosmotic therapy.

**Supplementary Information:**

The online version contains supplementary material available at 10.1007/s00701-021-04938-5.

## Introduction

Cerebral edema (CE), resulting from the abnormal accumulation of excess fluid in the intracellular or extracellular spaces, can co-occur with a heterogeneous group of neurological diseases, including stroke, infection, tumor, and traumatic brain injury (TBI), which leads to high morbidity and mortality [12, 26, 31]. CE generally causes a global elevation of intracranial pressure (ICP), while localized cerebral edema can lead to cerebral herniation syndromes with or without ICP elevation [28]. Especially CE caused by TBI is often associated with raised ICP, resulting in compression of brain structures and reduction of blood flow [26]. Current guidelines for treating CE with raised ICP include a combination of surgical and pharmaceutical therapy [7, 25, 27, 35].

Hyperosmotic therapy has been regarded as a pillar treatment for CE with raised ICP, with hypertonic saline and mannitol being the most used hyperosmotic agents. Despite being widely used, the disadvantages of hyperosmotic therapy include mainly acting on the normal brain region with limited effectiveness in eliminating the excess fluid in the edema area, along with other side effects [11, 16, 43]. The mechanism is to draw fluid out of the brain tissue into the vascular system by forming an osmotic gradient between the two. The excess fluid is extracted from brain parenchyma to the intravascular space by a high serum osmolality via intravenous infusion of hypertonic saline or mannitol, finally resulting in a reduction of brain volume and ICP [23, 39]. However, multiple clinical studies have shown that hyperosmotic therapy dehydrates mainly the non-lesioned tissue, and its capacity in eliminating excess water content in the edema region is rather limited [16, 21, 30, 43]. For the region involving CE, the edematous brain tissue squeezes the surrounding blood vessels, which reduces the cerebral blood perfusion resulting in the low efficiency of hyperosmotic therapy [34]. Moreover, the damaged blood circulation in the edema region causes vascular leakage [2], which also weakens the dehydration effect in the edema region.

In a previous study, we presented a novel electroosmosis based approach for CE treatment utilizing the brain tissue’s electroosmotic property [45]. Our approach is to induce electroosmotic flow (EOF) in the brain tissue by applying an external direct current to the head; as brain tissue is an electroosmotic material consisting of an electrical double layer (EDL) in the extracellular space, the cations driven by the electrical force move along the micro-channels through the extracellular space, which will pull the adjacent fluid to flow together by a viscous drag force, resulting in EOF [14, 40]. Intentionally placing an anode above the edema region and a cathode close to the subarachnoid space (SAS) allows edematous fluid to be driven out from the edema region into cerebrospinal fluid (CSF) circulation and finally absorbed to the cerebral venous system at SAS. Therefore, the electroosmosis based approach has a prominent efficiency in alleviating excess fluid in the edema region.

Given the limited efficiency of hyperosmotic therapy at the edema region, in contrast to the prominent efficiency of the electroosmosis based approach, we hypothesize that the latter can be designed to compensate for the deficiency of hyperosmotic therapy at the edema region, and thus, may improve treatment effectiveness for edema patients. For this, the electrode configurations in the electroosmosis based treatment can be designed to achieve intended EOF pathways, leveraging its effect by driving the edematous fluid to flow into the surrounding tissues more evenly and distributed to a larger region. Thus, the aim of this study is to investigate how to configure the electrodes of our previously proposed novel electroosmosis based edema treatment as a complement to hyperosmotic therapy.

A patient-specific head model is generated based on computed tomography (CT) images of a patient with localized edema. As the EOF direction is parallel to electric current flow, three configurations are designed with varying number of electrodes and locations. Current density and temperature are regarded as important criteria for brain damage [6, 9, 13, 33], which are kept at the same level in the numerical simulations of the three configurations.

## Methods

### Finite element (FE) head model development

#### Subject

**Fig. 1:**
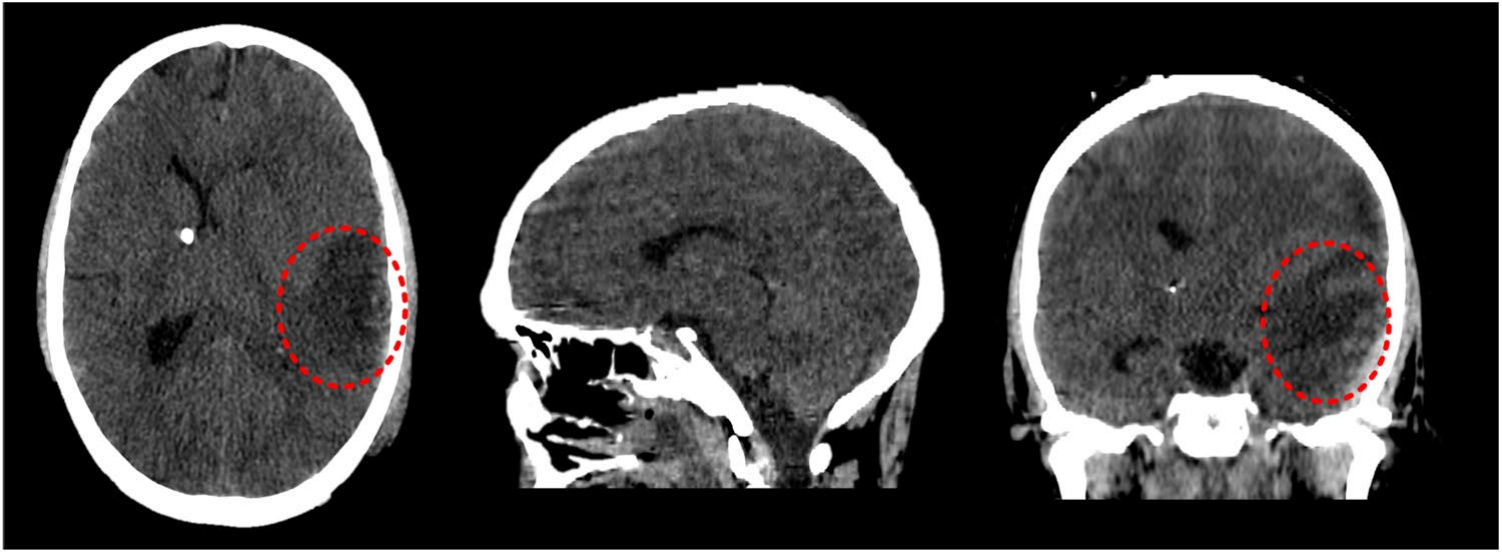
CT images of a patient with localized edema

The CT images of a patient with localized edema (Fig. 1) are retrieved from a previous study [44] to extract the geometry, based on which the patient-specific head model is generated. The CT images exhibit midline shift and compressed ventricular system, in which localized edema exists in the left hemisphere (right side of the images). According to the edema volume and water content (WC) quantified earlier [44], the excess fluid volume in the localized edema region is calculated to be 4.8 mL.

#### Patient-specific head model generation

**Fig. 2:**
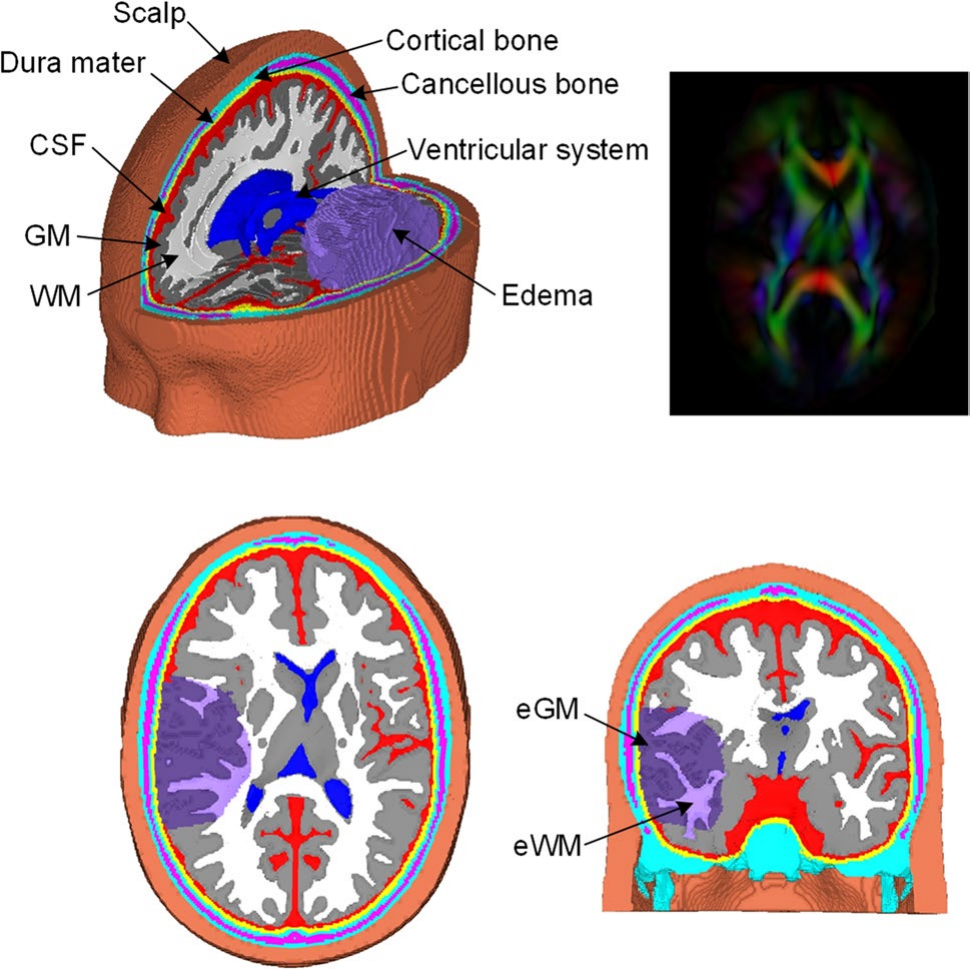
The generated patient-specific FE head model with personalized DTI incorporated, including eight components and a localized edema region

The patient-specific FE head model is generated via a personalization approach by morphing a baseline model developed and validated earlier [45]. The baseline model includes eight compartments, including the scalp, cortical bone, cancellous bone, dura mater, white matter (WM), gray matter (GM), CSF, and the ventricular system (Fig. 2). The model consists of 3.45 million hexahedral elements with a mesh resolution of 1 mm, which was generated using a smoothed-voxel algorithm. Details of the baseline model development and the personalization approach can be found in our earlier studies [32, 45].

**Fig. 3:**
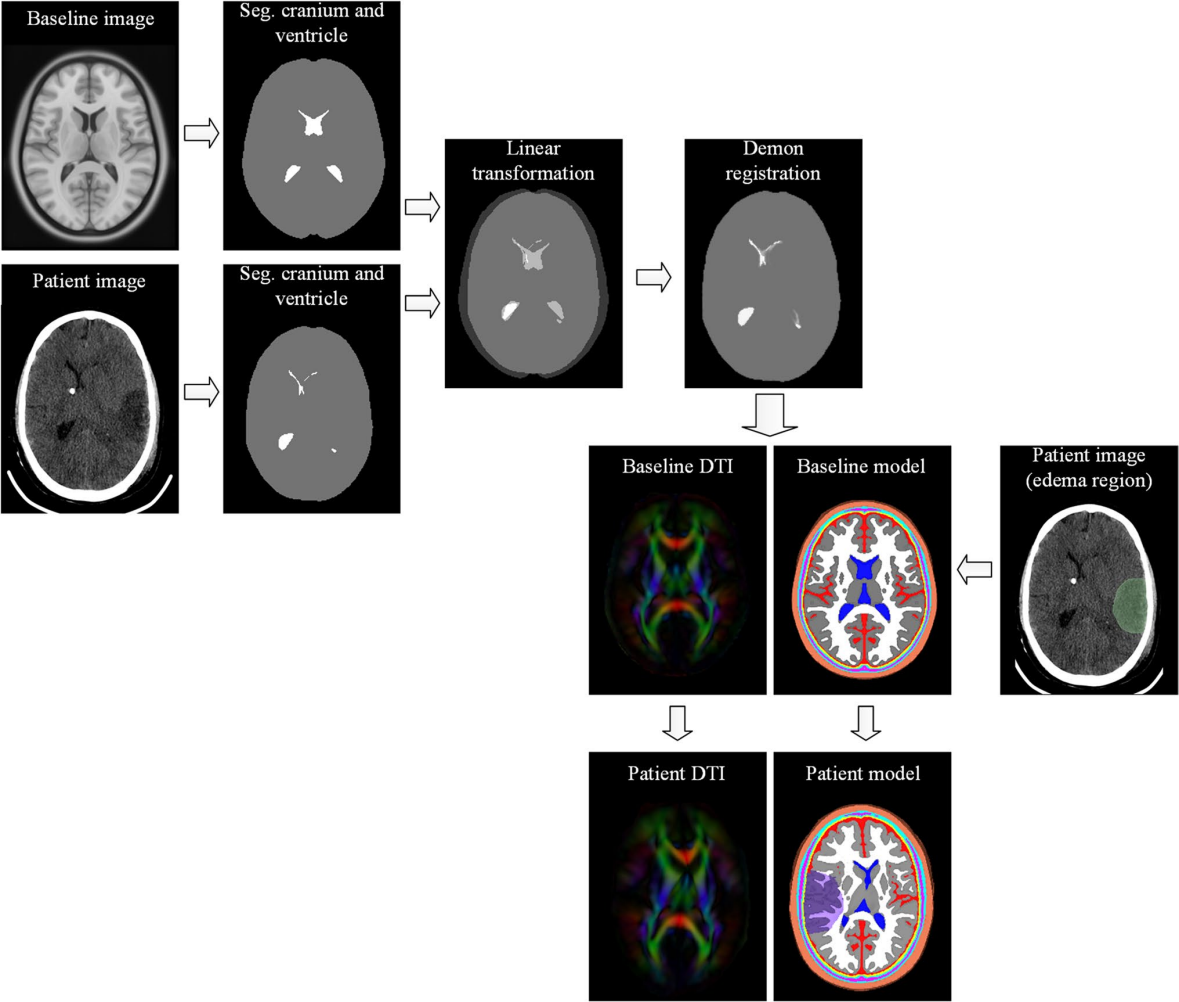
Overview of the personalization approach for generating the patient-specific head model and incorporating patient-specific DTI for anisotropic conductivity modelling. The CT images exhibit a localized edema in the left hemisphere (right side of the images)

The personalization consists of a registration pipeline (i.e., linear transformation and Demon registration) and morphing (Fig. 3). The registration pipeline starts from a linear transformation between the baseline image and the subject’s image for spatial alignment. Then, Demon registration of segmented cranial volume is performed (subject’s image as fixed image and baseline image as moving image) followed by a second Demon registration of the segmented ventricular system. Abovementioned two-step registrations result in two dense displacement fields capturing the geometrical difference between the baseline and the subject. Afterward, the two inverted displacement fields are applied to the baseline FE model and diffusion tensor image (DTI), resulting in a patient-specific head model and the corresponding DTI (Fig. 2). Finally, the edematous white matter (eWM) and edematous gray matter (eGM) are incorporated by grouping the FE elements based on the spatial location from the CT image.

### Electrode configurations

**Fig. 4:**
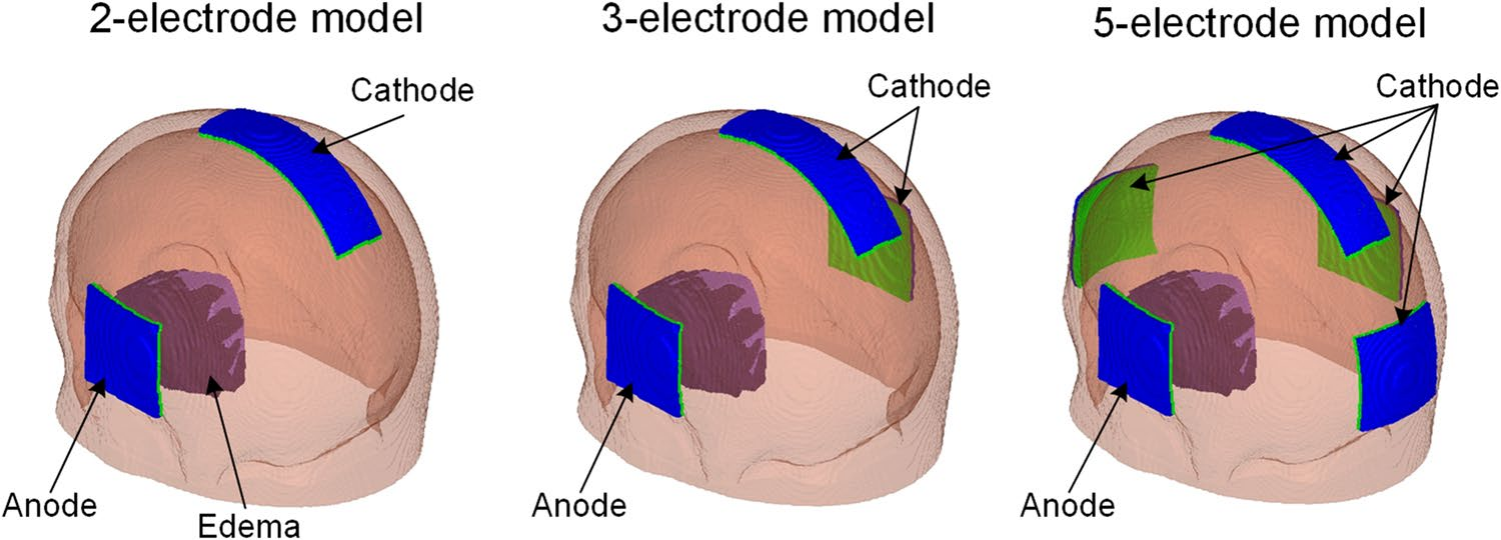
Three electrode configurations designed for a patient with localized edema, each model has one anode at the same location near the edema but with varying cathodes. The 2-electrode model has one cathode near SAS and one anode near the edema (left); the 3-electrode model has two cathodes (one near SAS and the other opposite to the anode) (middle); 5-electrode model has four cathodes near SAS, on the midforehead and occiput, as well as at the opposite position of anode (right)

Three electrode configurations (Fig. 4) are designed to investigate the distribution of electric field and EOF using the same patient-specific model. The same anode size (5 cm × 5 cm) is located close to the edema region, where excess tissue fluid is intended to be drawn out. In the 2-electrode model, a cathode (3 cm × 10 cm) is placed at the top of the head to facilitate the excess fluid to flow into SAS. In the 3-electrode model, besides a cathode (3 cm × 10 cm) located near the SAS, a second cathode (5 cm × 5 cm) is located at the opposite position of the anode intended to drive the edematous fluid to the surrounding tissue. In the 5-electrode model, besides two cathodes same as in the 3-electrode model, two additional cathodes (5 cm × 5 cm) are placed at the midforehead and occiput, aiming to facilitate edematous fluid to drive into the surrounding tissue more evenly and to a larger area.

A constant voltage of 15 V is applied to the anode in the 2-electrode model, whereas 12.9 V and 11.2 V are applied to the anodes in the 3- and 5-electrode models, respectively. The different voltages are chosen via a trial-and-error approach to achieve the same level of maximum current density and temperature to allow focusing on investigating the effect of electrode configuration on treatment outcome under the same safety level. The cathodes in the three models are set to 0 V, and all other external surfaces are assigned to be electrically insulated.

### Electroosmotic flow modelling

#### Electroosmosis theory

When a direct voltage is applied to the head model, the cations distributed in the extracellular space are driven by the electrical force to move along the micro-channels and pull the adjacent fluid by the viscous drag through the porous brain together. The EOF velocity is calculated by Helmholtz–Smoluchowski approximation [18, 22] as follows:

1 $$\nu =-\frac{{\varepsilon }_{r}{\varepsilon }_{0}\zeta E}{\eta }$$

where ν represents the EOF velocity, *ε*_*r*_ is the relative permittivity of the water (84.6), *ε*_0_ is the vacuum permittivity (8.85 × 10^−12^ F/m), *ζ* is the zeta-potential of brain tissue (− 22.8 mV), *E* is the electric field, and *η* is the water viscosity (6.4 × 10^–4^ Pa·s) at body temperature, in line with the parameters used in experiments for zeta-potential measurement [18, 19].

#### Electric field modelling

The electric field distribution across the head model is governed by Laplace’s equation under quasi-stationary conditions:

2 $$\nabla \bullet (-\sigma \nabla V)=0$$

where $\nabla$ denotes the gradient vector, $\sigma$ is the electrical conductivity of the tissue, and $V$ is the electric potential. The electric field, *E*, is calculated from the electric potential as follows:

3 $$E=-\nabla V$$

The current density, $J$, of volume conductor is determined by Ohm’s law:

4 $$J=\sigma E$$

The proportional anisotropic ratio algorithm [20] is employed to account for the anisotropic electrical conductivity in the WM based on the diffusion tensor extracted from the personalized DTI. A linear relationship between the eigenvalues of the diffusion tensor and the electrical conductivity tensor at each voxel is then used to calculate the eigenvalues of the electrical conductivity tensors:

5 $$\frac{{d}_{1}}{{\sigma }_{1}}=\frac{{d}_{2}}{{\sigma }_{2}}=\frac{{d}_{3}}{{\sigma }_{3}}$$

where ${d}_{1}$, ${d}_{2}$, and ${d}_{3}$ represent the eigenvalues of the diffusion tensor at each WM voxel, and ${\sigma }_{1}$, ${\sigma }_{2}$, and ${\sigma }_{3}$ denote the eigenvalues of the electrical conductivity tensor at the corresponding voxel. The volume constraint is combined to calculate the eigenvalues of the electrical conductivity tensor on the basis of keeping the volume of the anisotropic tensor and isotropic tensor same [46], i.e.,

6 $$\frac{4}{3}\pi {\sigma }_{\mathrm{iso}}^{3}=\frac{4}{3}\pi {\sigma }_{1}{\sigma }_{2}{\sigma }_{3}$$

where ${\sigma }_{\mathrm{iso}}$ denotes the isotropic electrical conductivity of WM or eWM.

### Temperature modelling

The Joule heating produced by the electric energy consumption *σ*|∇*V*|^2^ and metabolic activity cause temperature elevation, whereas blood perfusion results in heat dissipation. The bio-heat transfer model is governed by Pennes equation [9, 13]:

7 $$\rho {C}_{p}\frac{\partial T}{\partial t}=\nabla \left(\kappa \nabla T\right)-{\rho }_{b}{\omega }_{b}{C}_{b}\left(T-{T}_{b}\right)+{Q}_{m}+\sigma {\left|\nabla V\right|}^{2}$$

where $\rho$ is the tissue density, ${C}_{p}$ is the heat capacity of the tissue, $\kappa$ is the thermal conductivity, ${\rho }_{b}$ is the blood density, ${\omega }_{b}$ is the blood perfusion rate, ${C}_{b}$ is the heat capacity of the blood, *T* is the temperature, ${T}_{b}$ is the arterial blood temperature, and ${Q}_{m}$ is the metabolic heat source.

The convection of heat from the external surfaces of the head to the ambient is controlled by the following heat flux equation:

8 $$q=h\left({T}_{\mathrm{amb}}-T\right)$$

where *h* denotes the heat transfer coefficient (4 W/m^2^·°C), and ${T}_{\mathrm{amb}}$ represents the external ambient temperature (24 °C). The initial temperature values are set to 36.7 °C for head tissues and 24 °C for sponges and electrodes [9, 10, 13].

The electrical and thermophysical properties of the head tissues except WM and eWM are regarded as homogeneous and isotropic obtained from the literature [3, 5, 9, 10, 13, 17, 42, 45], in line with previous studies in the field of direct current stimulation (Table 1). For edema regions, the electrical and thermal conductivities of eWM and eGM are calculated based on the WC variation obtained from the quantitative WC maps (see Appendix A for details as Electronic Supplementary Materials).

**Table 1:** Electrical and thermophysical properties of head tissues

Tissue	Electrical conductivity σ (S/m)	Thermal conductivity κ (W/(m °C))	Metabolic heat source Q_m_ (W/m^3^)	Blood perfusion rate ω_b_ (1/s)
Scalp	0.435	0.39	363	1.43e − 3
Cortical bone	0.01	0.65	70	1.43e − 4
Cancellous bone	0.029	0.65	70	1.43e − 4
Dura mater	0.53	0.44	70	1.43e − 4
CSF	1.79	0.61	0	0
GM	0.333	0.565	16,229	1.33e − 2
WM	0.143	0.503	4517.9	3.70e − 3
eGM	0.359	0.566	8114.5	6.65e − 3
eWM	0.227	0.510	2258.95	1.85e − 3
Ventricular system	1.79	0.61	0	0
Electrode	5.99e7	31		
Sponge	1.4	0.3		

## Results

### Influence of electrode design on EOF distribution and treatment time

**Fig. 5:**
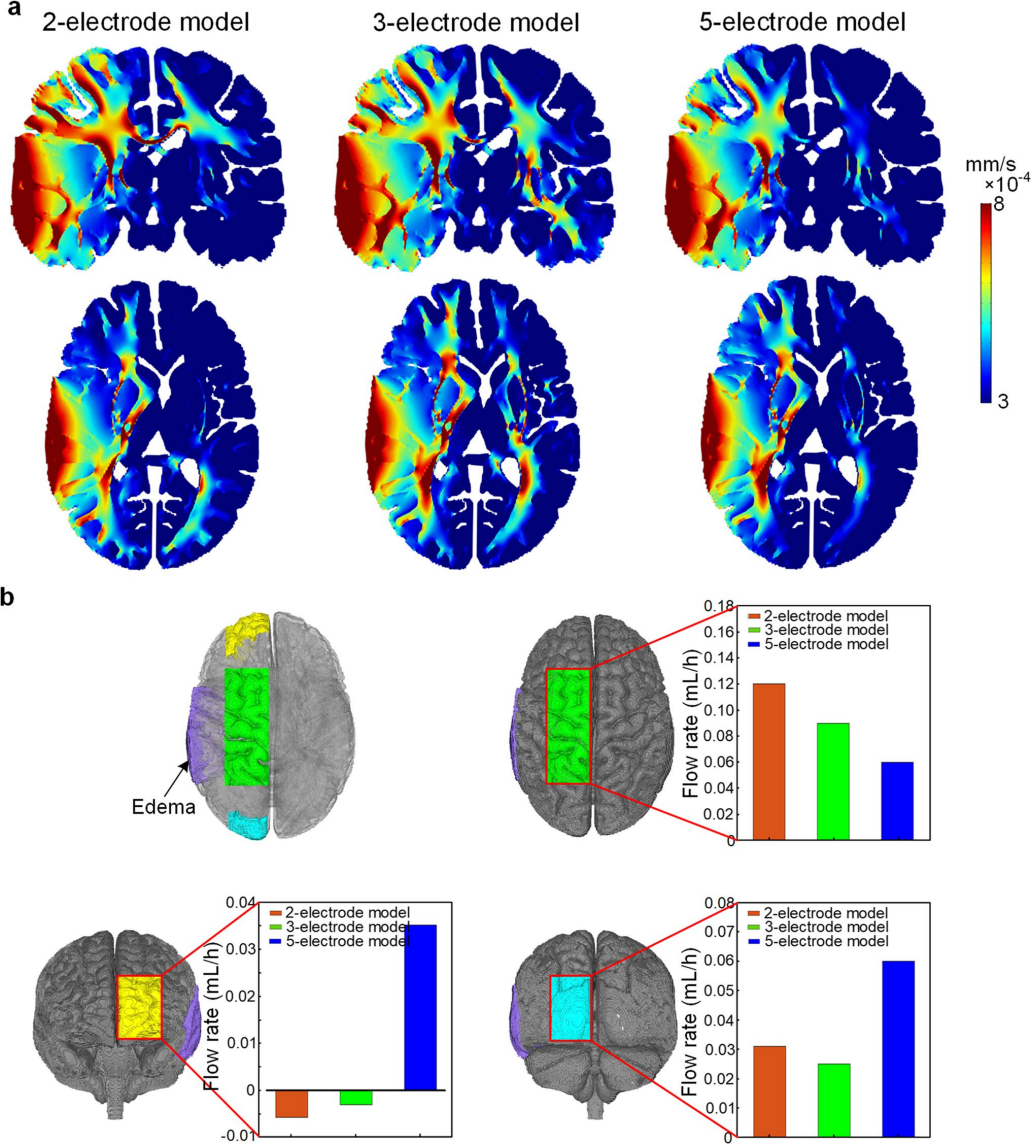
EOF velocity distribution and flow rates of ROIs for the three models. **a** EOF velocity distribution on the coronal and horizontal planes. **b** Flow rates of ROIs induced by an applied direct voltage. ROIs are located at top (green color), front (yellow color), and back (cyan color) areas of lesioned hemisphere, respectively

The average EOF velocity in the WM is higher than that for GM due to the lower electrical conductivity of the former (Fig. 5a). The EOF velocity distribution is similar inside the edema region among the three models, with highest value near the anode and decreases gradually to lower values than surrounding normal brain regions. For the normal brain region in the lesioned hemisphere, the 5-electrode model exhibits lower EOF velocity than the other two configurations as shown in the coronal plane. Moreover, the 5-electrode model also leads to the lowest EOF velocity in the contralateral hemisphere among the three models, suggesting this configuration has improved EOF focality and reduces the effect of EOF on the normal brain region than the other two designs.

Three regions of interest (ROIs) are located in the lesioned hemisphere to demonstrate the flow rates along different directions (Fig. 5b). For 5-electrode model, the flow rates on the front ROI (yellow color) and back ROI (cyan color) are higher compared to 2- and 3-electrode models, indicating more fluid is driven to flow along directions to the midforehead and occiput. In contrast, 2- and 3-electrode models exhibit the excess fluid mainly driven along directions from the edema region to SAS (green color). The quantitative results demonstrate that 5-electrode model allows driving the edematous fluid to flow into the surrounding tissues more evenly and distributed to a larger region.

**Fig. 6:**
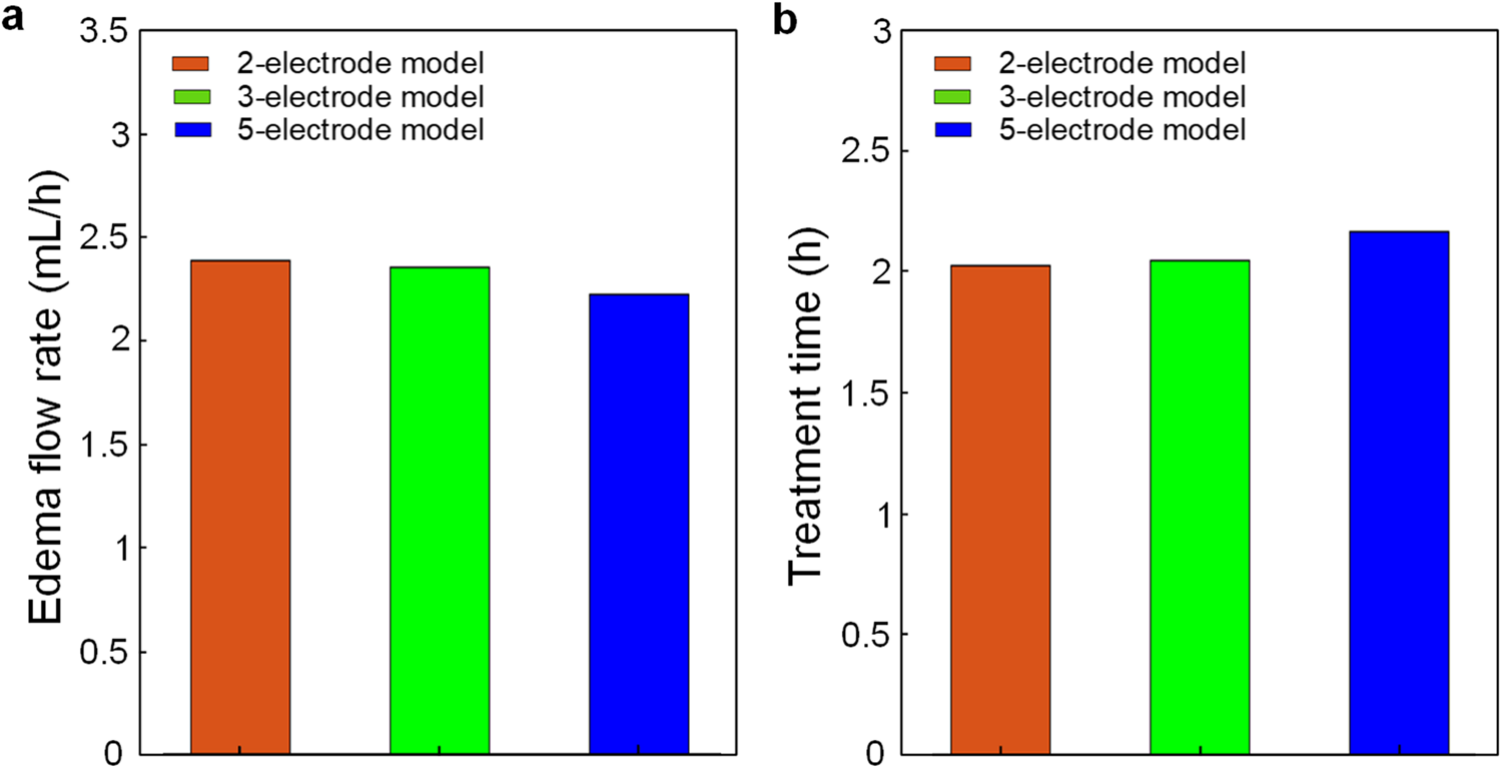
EOF flow rate and estimated treatment time for the three models. **a** Flow rate of EOF through the surface of edema region induced by an applied direct voltage. **b** Treatment time required to drive excess fluid out of the edema region

To evaluate the treatment time needed to draw excess fluid out of the edematous tissues, the flow rate is calculated by integrating fluid velocity over the surface of the edematous tissue with brain porosity of 0.2. As the edema region is underneath the anode, the flow rate (Fig. 6a) through the surface of the edema region induced by the direct current is up to 2.38 mL/h for the 2-electrode model, 2.35 mL/h for the 3-electrode model, and 2.22 mL/h for the 5-electrode model. Thus, given the volume of excess water for edematous tissue is 4.8 mL, the estimated treatment time (Fig. 6b) to drive excess fluid out of the edema region is around 2.02 h for the 2-electrode model, 2.04 h for the 3-electrode model, and 2.16 h for the 5-electrode model.

### Influence of electrode design on FOF direction

**Fig. 7:**
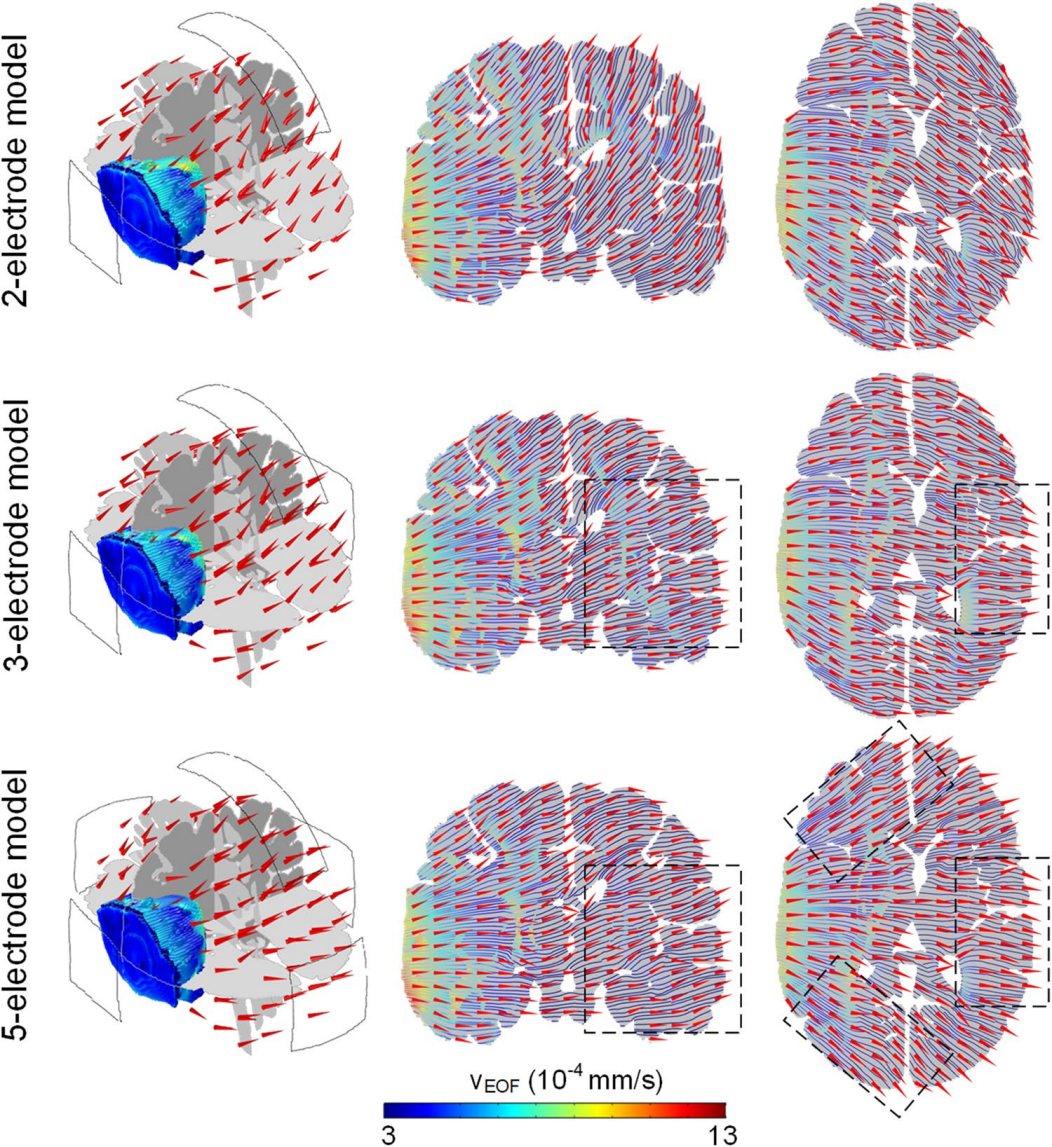
EOF direction inside the brain for all three configurations. The 1st column shows the EOF direction across the whole brain. The 2nd and 3rd columns represent the EOF direction and pathways on the coronal and horizontal planes. The red cone arrows show the EOF direction, and the streamlines represent the EOF pathways with color denoting the EOF velocity magnitude. The arrows in the black dotted line demonstrate the EOF direction variation among three configurations

The EOF direction across the brain for all three configurations is further analyzed (Fig. 7). For the 2-electrode model, the fluid is driven from the edema region to SAS parallel to the direction of electric current flow from anode to cathode. For the 3-electrode model, part of the edematous fluid is driven along the direction from the edema region to the contralateral hemisphere due to an additional cathode located at opposite of the anode. For the 5-electrode model, due to the existence of four cathodes, edematous fluid is partially driven from edema to SAS, and part of the fluid is driven to flow along the direction from the edema region to the contralateral hemisphere, midforehead, and occiput, respectively.

### Safety evaluation: current density and temperature

**Fig. 8:**
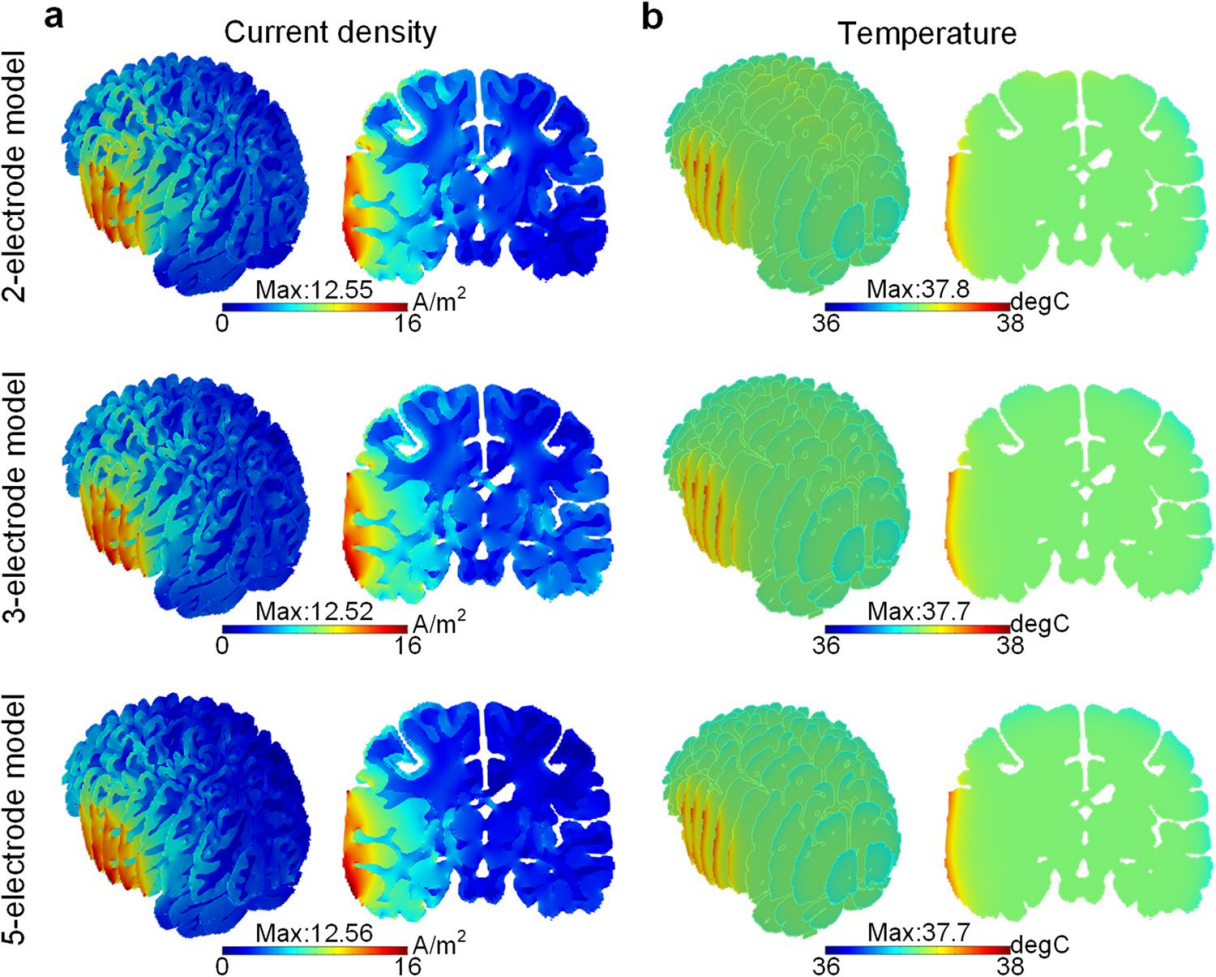
Distribution of current density **a** and temperature **b** across the brain and on the coronal plane for the three configuration models

The distribution of current density and temperature across the brain for the three configurations are investigated (Fig. 8). Significantly high current density and temperature are observed in the edema region underneath the anode. The maximum current density (calculated as the 99^th^ percentile) in the entire brain is at the same level for all three models, being 12.55 A/m^2^, 12.52 A/m^2^, and 12.56 A/m^2^ for the 2-, 3- and 5-electrode models, respectively. Moreover, the maximum temperature induced by an applied electric field in the three models is also similar, and a maximum value is 37.8 °C for all three designs, which is within the safety range.

## Discussion

In this study, the possibility of configuring an electroosmosis based treatment as a complement to hyperosmotic therapy for CE is investigated, and the findings demonstrate that the edematous fluid can be driven out of the edema region to flow into the surrounding tissues evenly by designing the electrode configuration. Of the three configurations investigated, the 5-electrode design shows improved EOF focality with a reduced potential effect on normal brain region, and better directionality allowing driving the fluid from the edema region into the surrounding tissues more evenly where it can be absorbed by a larger volume of tissue during hyperosmotic therapy. Thus, when the excess fluid is driven out from the edema to the surrounding normal brain region, it can then be treated using hyperosmotic therapy. Based on this, the limited efficiency of hyperosmotic therapy on the edema region is compensated by the prominent efficiency of the electroosmosis based treatment for CE.

Since the CE caused by TBI is often accompanied by raised ICP, which has a high risk of causing irreversible brain injury and death [26], hyperosmotic therapy is usually employed to reduce the raised ICP rapidly, which is non-specific and targets towards alleviating the effect of edema. In contrast, the electroosmosis based treatment focuses on the alleviation of the abnormal fluid accumulation, leveraging its effect by driving the edematous fluid out of the edema region. Especially for smaller channels, when the fluid flow is low due to higher hydrodynamic resistance, the EOF induced by an applied direct current is most significant [8]. In the present study, the excess water volume for the patient with localized edema is calculated with a value of 4.8 mL, whereas the brain volume decreased by around 8 mL with a 20-min infusion of 40 mL of 20% saline or a 1.5 g/kg bolus infusion of mannitol [30, 43]. Based on this, when the 5-electrode configuration is employed to drive the excess fluid in the edema region to flow into the normal brain tissue evenly, the excess fluid can be extracted from the brain parenchyma to the intravascular space by hyperosmotic therapy. In addition, the continuous hyperosmotic therapy and electroosmosis based treatment might be used simultaneously to drive the excess fluid out of the edema region then absorbed into intravascular space directly by osmotic gradient.

For the 2-electrode model, the excess fluid is mainly driven to flow along the direction from the edema region to SAS directly. In the 3-electrode model with an extra cathode placed at the opposite position of the anode, part of the edematous fluid is driven to flow along the direction from the edema to the contralateral hemisphere. For the 5-electrode model, due to the existence of four cathodes, edematous fluid is driven to flow along the directions from the edema region to SAS, the contralateral hemisphere, and midforehead, as well as occiput, respectively. Therefore, the 5-electrode model is more suitable to drive the edematous fluid into the surrounding tissues evenly. The flow rate of the edematous fluid volume being driven out is predicted to be 2.22 mL/h for the 5-electrode design, which is comparable to the 2-electrode and 3-electrode designs indicating a similar efficiency drawing edematous fluid from the edema region. In addition, the normal tissues for the 5-electrode model exhibit a lower and more evenly distributed velocity of EOF compared to the other two models, whereas three models show a similar distribution of EOF velocity in the edema region, indicating that the electrode configuration for the 5-electrode model could improve the EOF focality and reduce the effect of EOF on the normal tissues. Based on the above analysis, the electroosmosis based treatment shows the capability of driving edematous fluid into the surrounding tissues evenly, and thus could be considered a complement to hyperosmotic therapy to potentially improve the treatment efficiency for edema patients.

As the EOF results from the movement of the charged ions under an applied electric field [15, 18, 19], the EOF direction is mainly determined by the electrode configuration, which is parallel to the direction of the current flow from anode to cathode. The direct current applied to the anode flows across the brain along the direction from anode to cathodes, indicating that the induced EOF will be directed to the regions underneath the cathodes. Therefore, for the 2-electrode model with a cathode located near SAS in line with our previous study [45], the activated region with high EOF velocity is mainly located under and between the edema region and SAS. For the 3-electrode model and 5-electrode model, due to the existence of extra cathodes, the excess fluid in the edema region is driven to flow along direct current pathways to surrounding normal brain tissue, resulting in the reduction of abnormal accumulation of fluid within the edema region. Moreover, the predicted average EOF velocity in the WM is higher than that in the GM due to the lower electrical conductivity of the former; given the swelling extent in GM is usually lower than WM [1, 29, 44], the electroosmosis based treatment results in a better alleviation of the abnormal fluid accumulation in the WM.

The capability of inducing fluid flow in brain tissue cultures [36–38] and in rat brain [14, 15] by direct current has been confirmed in experiments based on the brain’s electroosmotic property. Moreover, a significant stimulation-polarity-specific fluid and solute movement is induced when applying direct current to endothelial monolayers, suggesting the electroosmotic property of the brain [8]. An essential condition to induce EOF inside the brain is the narrow channels with charged walls such as the extracellular space [18, 36–38]. Thus, the electric field mainly induces EOF in the brain parenchyma. Based on this, the electroosmosis based treatment mainly affects the interstitial fluid transport in the extracellular space of the brain parenchyma. Moreover, in vivo two-photon imaging of small fluorescent tracers showed that CSF moved along paravascular spaces that surround arteries and that interstitial fluid was cleared along paravenous drainage pathways [24, 41]. Therefore, when the excess fluid and solutes are driven out from the edema region to the surrounding brain regions by electric field, they can be cleared along paravenous drainage pathways via the glymphatic system. Finally, the excess fluid will be extracted from paravascular space to the intravascular space by a high serum osmolality, resulting in a reduction of brain volume and ICP. Whether or not this approach may alleviate cellular swelling (e.g., cytotoxic edema) could be further investigated.

Given that the current density and temperature are regarded as important criteria for brain damage [6, 9], the applied voltage for three models is adjusted to achieve the same level of the maximum current density and temperature. According to the animal experiments in the field of the direct current stimulation, the minimum current density induced on the cortical surface for detected damage was 12 A/m^2^ [6, 33], whereas the damage threshold for maximum electric field is about 61 V/m for a current duration of 20 min [4]. In this study, for the 5-electrode model, the maximum current density and electric field for the brain tissue are 12.56 A/m^2^ and 34.9 V/m, respectively, which is close to the above safety range. For the temperature variation caused by the electric energy consumption, the maximum temperature for the 5-electrode model with adjusted applied direct voltage is 37.7 °C, which is below 38.0 °C within the safety range [10, 13]. Since there is no specific threshold for brain damage caused by direct current, further studies need to be performed to fill the knowledge gaps in safety criteria. In addition, as the EOF velocity is proportional to the electric field and the EOF flux is linearly dependent on current density, it is reasonable to expect that brain damage could be avoided by reducing the induced EOF velocity with a lower applied direct voltage.

The results of this modelling study show the promise of developing the electroosmosis based approach as a complement to hyperosmotic therapy for CE treatment; the efficiency of the proposed combined approach is yet to be confirmed via experimental studies, e.g., rat and pig experiments, in which CE will be generated by controlled cortical impact or cold injury and then treated based on the above design. The results obtained from animal experiments will be transferred to human head models by scaling laws to further optimize electrode configuration and magnitude of the applied direct current.

Some limitations need to be mentioned for this study. Since weaker direct current is usually used to modulate cortical activity, whether a higher direct current density used in the present study may influence the network function should be further investigated. Further, during electroosmosis based treatment, when excess fluid is driven out of the brain parenchyma, brain deformation is expected to occur, and the electrical parameters of tissues will change, indicating that the predicted treatment time may be under- or overestimated. Finally, the predicted treatment time reported here represents how much time needed to draw the excess fluid out of the edema region to the surrounding tissue, which does not account for potential dynamic development of edema.

## Conclusion

In conclusion, the possibility of developing electroosmosis based treatment as a complement to hyperosmotic therapy for CE is investigated, and the results demonstrate that the limited efficiency of hyperosmotic therapy on the edema region can be compensated by the prominent efficiency of the electroosmosis based treatment for CE. The excess fluid in the edema region can be driven to flow along different directions, showing the capability of changing the EOF pathways by designing the electrode configuration.

## Supplementary Information

Below is the link to the electronic supplementary material.

Supplementary file1 (PDF 373 KB)
