# Supplementary material for: Designing electrode configuration of electroosmosis based edema treatment as a complement to hyperosmotic therapy
Source: Acta Neurochir (Wien). 2021 Jul 22;163(9):2603–14. doi: 10.1007/s00701-021-04938-5 (PMC8357759; doi:10.1007/s00701-021-04938-5)
Supplement: Supplementary file 1 — Supplementary file1 (PDF 373 KB) [file 701_2021_4938_MOESM1_ESM.pdf]

Designing electrode configuration of electroosmosis based edema treatment as a complement to hyperosmotic therapy

Teng Wang[1]*, Svein Kleiven[1], Xiaogai Li[1]

[1] *Division of Neuronic Engineering, Department of Biomedical Engineering and Health Systems, KTH Royal Institute of Technology, Huddinge, Sweden.*

*Corresponding Author:

Teng Wang
Email: tenwan@kth.se

Appendix A: Conductivity calculation for edema tissues

Since the excess fluid of CE mainly comes from vascular leakage, we assume the increased electrical and thermal conductivity of the edema region as a combination of brain and plasma conductivity. Given the brain conductivity is proportional to the tissue water percentage [2], we consider the ratio of WC in edema region (e.g., eWM and eGM) and contralateral hemisphere (e.g., WM and GM) as the algorithm to estimate the edema conductivity. The average WC in the edema region and contralateral hemisphere is calculated based on the quantitative WC maps (Fig. 9) of the patient obtained from an earlier study [3], and the quantified WC for the patient is presented in Table 2. Then the electrical and thermal conductivities are calculated according to the WC ratio between edema region and contralateral hemisphere. For example, as WC increases from 73.8% to 81% in eWM for the patient, the electrical and thermal conductivities of eWM are estimated as 0.227 S/m and 0.510 W/(m· °C) (91.1% WM and 8.9% plasma), respectively, where electrical and thermal conductivity of plasma is 1.09 S/m and 0.58 W/(m·°C) [1]. The estimated thermal and electrical conductivities in WM, GM, eWM, and eGM for the patient model are summarized in Table 1.

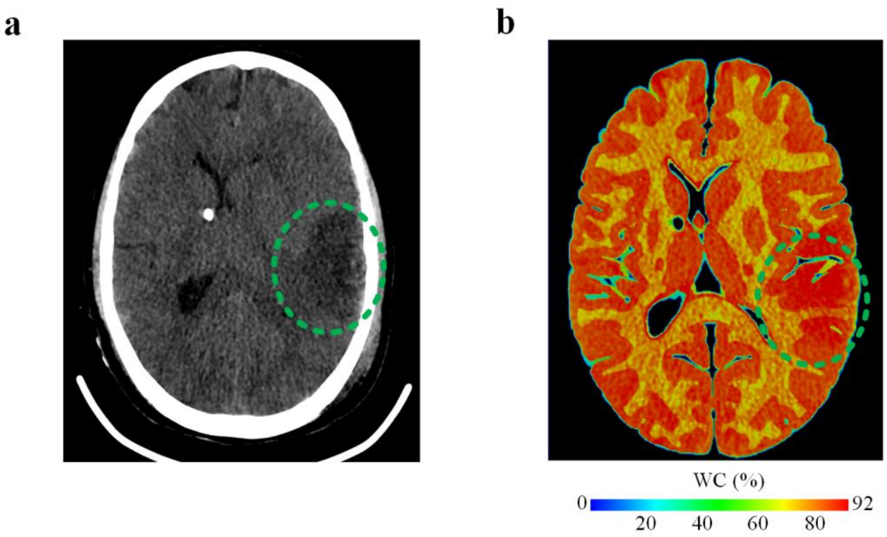

Appendix Fig. 9. a Representative horizontal slices from the CT images of the three patients. **b** Quantitative WC maps on the horizontal slices for the patient.

Appendix Table 2. WC, electrical conductivity and thermal conductivity in edema region (eWM and eGM) and normal tissues (WM and GM) for the patient-specific head model

	LE			
	WM	eWM	GM	eGM
WC (%)	73.8	81.0	85.0	88.1
σ (S/m)	0.143	0.227	0.333	0.359
κ (W/(m• °C))	0.503	0.510	0.565	0.566

*LE - patient with localized edema; WM - white matter; eWM - edematous white matter; GM - gray matter; eGM - edematous gray matter; WC - water content.

References

1. Gabriel C (1996) Compilation of the dielectric properties of body tissues at RF and microwave frequencies. King's Coll London (United Kingdom) Dept of Physics,
2. Harting MT, Smith CT, Radhakrishnan RS, Aroom KR, Dash PK, Gill B, Cox Jr CS (2010) Regional differences in cerebral edema after traumatic brain injury identified by impedance analysis. Journal of Surgical Research 159:557-564
3. von Holst H, Li X, Kleiven S (2012) Increased strain levels and water content in brain tissue after decompressive craniotomy. Acta Neurochir 154:1583-1593
